# General collections demography model with multiple risks

**DOI:** 10.1057/s41599-025-05325-6

**Published:** 2025-06-21

**Authors:** Josep Grau-Bové, Miriam Andrews

**Affiliations:** University College London, London, UK

**Keywords:** Complex networks, Science, technology and society

## Abstract

This paper presents an Agent-Based Model (ABM) with Monte Carlo sampling, designed to simulate the deterioration processes within a population of objects over time. The model incorporates damage functions with the risk parameters of the ABC framework to simulate adverse events. As a result, it combines continuous and probabilistic degradation. This hybrid approach makes it possible to study the emergent behavior of the system and explore the range of possible lifetimes of collections with cultural value or scientific interest within galleries, museums, archives or libraries. A toy application of the model is tested with paper, with the main outcome of the model being the decay in condition of a collection as a consequence of all the combined degradation processes. The model is based on six hypotheses that are described for further testing. This paper presents a first attempt at a universal implementation of Collections Demography principles, with the hope that it will generate discussion and the identification of research gaps.

## Introduction

The concept of Collections Demography, developed by Strlič et al. (2015), originated from the need to develop evidence-based management strategies for historic collections. In the context of libraries and archives, users are primarily concerned with textual information, which if sufficiently lost to degradation, may cause the collection item to be categorised as unfit for use. To understand the mechanisms behind this deterioration further, researchers have developed damage functions (mathematical models of material change) that combine aspects of material degradation, use, and material attributes important for user interaction with heritage (Strlič et al. 2013). These functions are based on data from paper degradation experiments and real collections. By treating the collection as a population of objects with different characteristics and combining this with damage functions that suggest the deterioration over time, the effects of various management strategies can be modelled to provide informed decisions based on predicted outcomes. Collections demography has been used for the evaluation of scenarios for managing storage environments and levels of access for different types of library and archival paper (Duran-Casablancas et al. 2021). Michalski (2013) also proposed a collection-wide model focused on mechanical damage to paintings. Clearly, the collections demography principles have demonstrated advantages in the sustainable management of collections. However, their application has so far been restricted to very specific collection types. The question remains as to whether collections demography could be extended to any collection, from coins to churches?

Recent efforts by the Department for Culture, Media, and Sport (DCMS) of the UK Government to develop a framework for valuing culture and heritage capital have highlighted the significance of damage functions in this context (Clark 2021). The goal of the “Culture and Heritage Capital Programme" is to create a formal approach to value culture and heritage assets, which will ultimately inform decision-making processes in the public and private sectors. Sagger and Bezzano (2023) have put forward a proposal to integrate economic valuation methodologies with degradation rates to measure the welfare impact of interventions that halt the loss or deterioration of cultural and heritage assets. This requires an ability to calculate how any intervention, to any collection, will impact its lifetime.

The success of these policy initiatives is predicated on the existence of damage functions that have sufficient accuracy in predicting the degradation of most relevant collection materials. This is complicated for several reasons. Firstly, there are only a handful of materials for which damage functions have been produced that are at an advanced enough level to generate lifetime estimates, e.g., photographs (Fenech et al. 2012), paper (Strlič et al. 2015), and PVC (Rijavec et al. 2023). Secondly, damage functions that predict chemical degradation are only a part of the picture. There are also other types of ongoing processes that affect most materials; in the case of paper or canvas, for example, items may undergo physical changes related to RH fluctuations, mechanical damage from wear generated by repeated handling or cleaning, or biological damage from decomposition processes associated with mould. Different material typologies have varying sensitivities to these types of processes that need to be accounted for within a modelling approach. Lastly, as it is well known, heritage degrades through both continuous deterioration and catastrophic events, like fire, theft, or flooding (Michalski 1990), necessitating a holistic risk approach, i.e., considering all potential risks comprehensively and together.

Several risk assessment approaches with quantitative and holistic aspects are used within preventive conservation practice and academic research. The ABC framework is well-established and provides a structured approach to risk management, focusing on specific risk factors and their impacts (Michalski and Pedersoli Jr. 2016, Michalski and Karsten 2018). The framework is now available as an online program, making it accessible and user-friendly (COC/FIOCRUZ et al. 2022). However, rates and probabilities for processes are based on user estimates (with guidance provided by expert knowledge), meaning that the framework does not make use of available damage functions and may not incorporate relevant statistical data. HERIe, another online digital platform, uses quantitative assessment tools based on modelled deterioration processes to evaluate risks to collection objects (Kupczak et al. 2018). While HERIe provides robust assessment, it relies heavily on predefined modules that are not combined into an overall model of risk. At present, operational modules mainly focus on risks that take environmental data as input, e.g., relative humidity, temperature, and Lux, although expansions to other types of risk, for example, fire (Bratasz and Berger 2024), have recently been made. Crucially, neither of the two approaches described above are able to output impact in terms of changes to the lifetime of collection objects.

To accomplish the ambitious vision of the “Culture and Heritage Capital Programme", the field of heritage science must come together to develop more comprehensive damage functions for continuous deterioration processes and also to establish better understanding about the levels of risk from other hazards that cultural heritage institutions face, while ensuring these interrelated processes work together effectively. However, this ideal may be years away. This paper proposes that, in the meantime, using collections demography principles in with conjunction with agent-based models (ABMs) (or other similar statistical models) is the key to combining the different types of degradation processes that affect collections, as well as handling the uncertainties within the system.

Agent-based models can simulate changes that occur to individual agents (e.g., objects in a collection) over time, providing a granular view of deterioration processes. By modelling interactions between agents, the model can reveal complex system dynamics that might not be apparent in more static models. While ABMs can be computationally intensive to develop, once established it is relatively easy to incorporate various types processes, e.g., risk scenarios and damage functions, allowing for a comprehensive and adaptable model. The use of probability distributions within their risk sampling procedure allows for the modelling of uncertainty and variability within deterioration processes. However, it is sometimes a challenge to obtain the high-quality, detailed data that is necessary to accurately parameterise these models. Furthermore, due to the lack of comparative datasets, ensuring a model’s accuracy and reliability through validation can also be difficult.

Nonetheless, in other fields, agent-based models have been widely used to study the ageing of different types of “populations", from the literal ageing of patients (Spijker et al. 2022), to survival rates during clinical trials (An 2001), to the mechanical breakdown of engineering assets as diverse as pipeline infrastructure (Li et al. 2020), structural components within civil engineering (Guo et al. 2020), and maritime vessels (Liu and Frangopol 2018). All these examples are partial analogies to heritage collections. They display some of the key features similar to the deterioration system of cultural heritage, but rarely all of them. The characteristics that define the system that is unique to cultural heritage can be considered as follows:

1. There is a finite population
2. Each agent in the population has a key property (such as condition or value) that decreases over time
3. The decreasing property is affected by gradual rate-processes as well as probabilistic accidents
4. The process of decay is extremely slow, of the order of centuries
5. The slowness of the process is such that it cannot be assumed that the decreasing property (condition or value) will be defined in the same way in the future. In other words, societal change is of a similar time-scale than the physical process.

As far as the authors are aware, there are not any analogous or similar systems in any other field that fulfill all these criteria, and certainly none that have already been modelled using computational simulation. Hence, the application of ABMs to heritage collections provides a new modelling scenario and will, inevitably, be full of research challenges. This paper presents a first attempt at investigating the problem, with the hope that it will generate discussion and the identification of research gaps. To aid discussion, every time a new hypothesis is introduced, it will be noted and marked with an index like “H0".

### An operational definition of lifetimes

To calculate the lifetimes of heritage objects, a definition of unacceptable degradation is required. A limit that is commonly adopted is the threshold beyond which the social function of an artifact changes fundamentally, i.e., the ‘end-of-life’ (Strlič et al. 2013). For example, a book may be degraded to the point it cannot be handled by readers, or a tapestry may be faded to the point of not being decipherable. Another alternative definition can be understood as the point where value loss is such that a clear need for investment or action emerges. The word “operational" signifies clearly that this threshold is meant to enable informed management.

A reductive approach is necessary in deciding a threshold because it helps to inform better decision-making. However, it is adopted with the full knowledge that any quantitative straight line drawn over a social continuum is, of course, a fiction. In many instances, it is difficult to approximate this limit of unacceptable degradation because many stakeholders will perceive the damage differently (Taylor and Stevenson 1999). What is damage in some contexts is patina in others. Social and curatorial contexts influence the perception of age and loss (Grossi and Brimblecombe 2004). The fundamental hypothesis of collection demography is that these thresholds are definable for a wide diversity of heritage typologies (H1). Only one or two decades of research in perception and the social value of heritage may bring us closer to workable definitions of unacceptable change for all the useful cases.

**Fig. 1:**
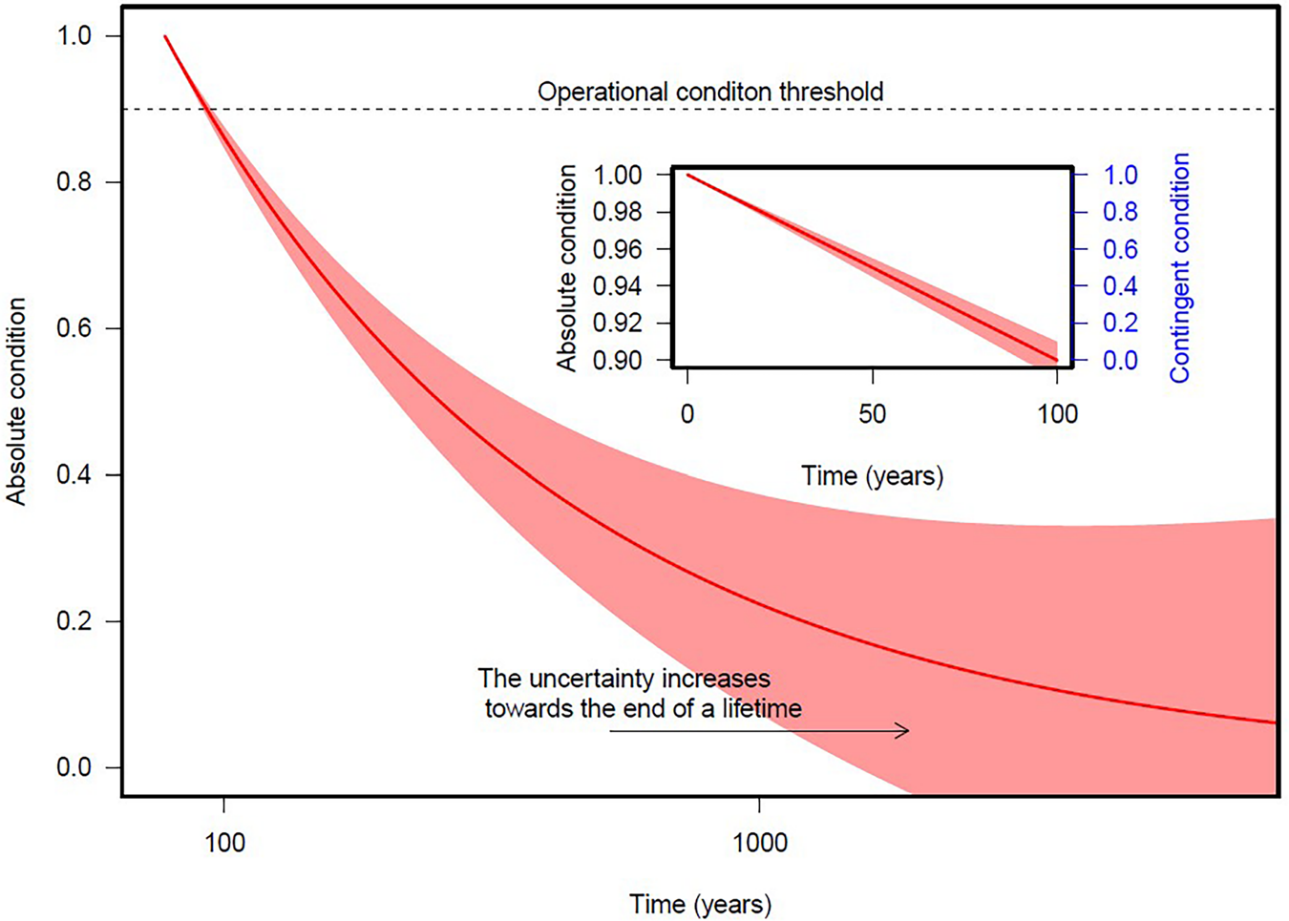
Illustrative graphic demonstrating that condition decays in an unknown and unpredictable way, but the crux of decision-making is focused on a small time window at the start of the process.

These issues may be set aside in the pursuit of a practical solution. Within this scenario, it is necessary to assume that a condition state can be defined for each artifact: a quantity that decreases from 1 to 0 as the artifact ages. This is purely a theoretical construct and can be referred to as the absolute condition of the object. In practice, there is no need to attempt to model the entire lifetime of an object, from condition 1 to condition 0, for two reasons. Firstly, it is likely than in a lifetime of hundreds of years, the way society sees an object will change more than the object itself. Secondly, it is likely that as an object approaches the end of its lifetime, its function will change, for example through deaccessioning, joining a handling collection, or being forgotten. When this happens, the artifact will drop out of the management system we are attempting to model. To avoid the great uncertainty that these processes will bring to modelling, it is sensible to refrain from predicting the decay of absolute condition. Let us consider instead that, at some point early in the lifetime, the absolute condition will hit the operational limit that will trigger an action or investment. For example, as commonly assumed, a museum will worry when fading begins to be visible, rather than when a watercolor is absolutely white. As Fig. 1 represents, this point in time helps us define a shorter, less uncertain lifetime. We define a new contingent condition as the one that becomes 0 when the absolute condition hits the operational threshold.

We should note that in this simple framework we are introducing another important dynamic hypothesis: that the probability of an action in response to condition increases in a step-wise manner when condition decreases. We can set up a threshold of unacceptable condition with ease when there is a condition above which most actors perceive a change or decide to act upon it. The sharper this change in perception, the more realistic it is to define an operational threshold. While a step-wise response is not essential, it is highly desirable. We may call this the “sharp response hypothesis" (H2). In some cases, for example, the number of observers who see a surface as visibly dirty has been found to increase sharply at 5–10% area coverage by particulates. However, this behaviour may not be universal.

## The model

### Modelling degradation rates with damage functions

**Fig. 2:**
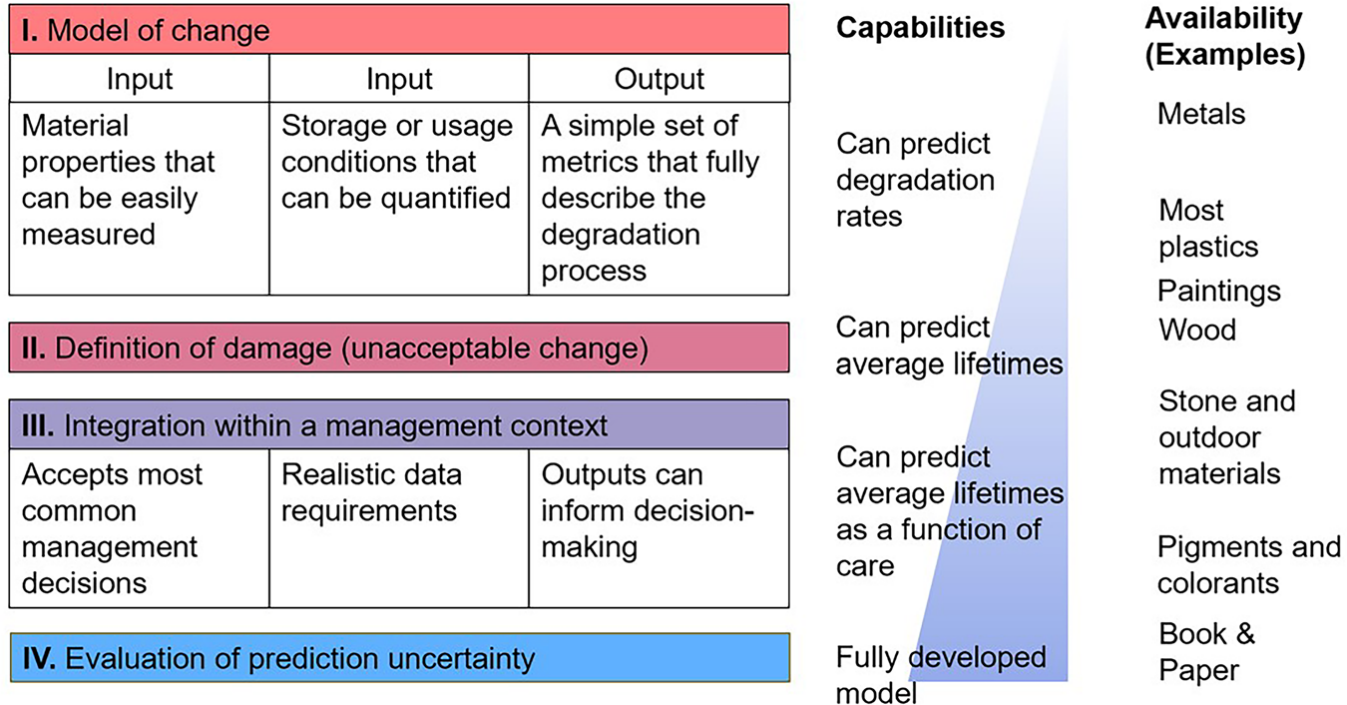
Diagram showing a summary of the capabilities of damage functions depending on their level of development.

The general demographics model relies on the existence of damage functions that provide the decay rate of key properties (color, surface recession, mechanical strength, degree of polymerisation, etc.). It is necessary to assume that these key properties are directly proportional to the absolute condition of an object (H3). The model also requires a definition of the operational threshold for these properties as an input. However, not all damage functions are at the required level of development. For the purpose of clarifying the state of development of damage functions for different materials, we have classified them into four levels below (Fig. 2):

A Level I damage function includes a model of change with well-defined inputs and outputs, identifying which input parameters are critical and which can be ignored. There are models at this level of detail for many materials, for example, for metals (Thickett et al. 2013), cellulose acetate (Ahmad 2022, King et al. 2020), or paintings on canvas and wood (Jakieła et al. 2008, Mecklenburg et al. 1998, Rachwał et al. 2012, Rachwał et al. 2012). At this level, we understand the physicochemical processes at play.

A Level II damage function can estimate lifetimes, because the physicochemical model is paired with a definition of damage derived from stakeholder preferences and value judgments. For example, we know at which point fading is just noticeable. It is also possible to define levels of stone recession where essential detail is lost. Many heritage materials such as plastics (Rijavec et al. 2023), paintings (Bucklow 1999, Zhang et al. 2023), and wooden objects (Kupczak et al. 2018) are nearing level II, although the definitions of damage for most remain open to debate.

Level III damage functions have aligned their input with management decisions. They contain inputs that correspond with what a manager would know. The functions that predict fading are good examples (Fenech et al. 2012), as they relate to Lux and light spectra of common sources.

The most advanced Level IV damage functions also evaluate uncertainty, offering lifetime estimates with a margin of error. Currently, only the damage functions for paper have reached this level (Strlič et al. 2015).

The model presented here requires at least Level II damage functions.

### Definition of adverse events

This model takes advantage of the ABC framework for risk assessment (Michalski and Pedersoli Jr. 2016). Consequently, adverse events in the model are defined by three parameters, each characterized by a level of uncertainty.

- **Mean Time (A):** This parameter defines the mean time before an adverse event occurs. It is also expressed as a range, reflecting the uncertainty in the timing of event occurrences.
- **Extent of Impact on Condition of Objects (B):** This parameter quantifies the degree to which the condition of affected objects is reduced following an adverse event. It is given as a range, such as 0.2 to 0.4.
- **Fraction of Collection Affected (C):** This parameter represents the proportion of the population or collection of objects that is impacted by an adverse event. It is expressed as a range, such as 0.01 to 0.2, indicating the uncertainty in the extent of the event’s impact.

The application of the ABC risk assessment model in this context approaches the limit of its suitability and intended use. This ABC model is primarily designed to facilitate decision-making by comparatively identifying and prioritising the most significant risks in a semi-quantitative scoring system, rather than delivering precise risk predictions (though it has been used in this way before by Michalski and Karsten (2018)). The authors of this type of risk assessment have cautioned users about its limits from the very start. The words of Robert Waller in 1994 are still true: “Currently, the information required to produce accurate estimates of the magnitude of many risks is lacking. Nevertheless, simply attempting the exercise among a group of collections care staff produces several valuable results" (Waller 1994). Another critical hypothesis is, therefore, that the ABC method can eventually become the basis of quantitative forecasts (H4). As we shall see, the simulation model presented in this paper can help evaluate this hypothesis.

Table 1 contains several examples of some of the types of adverse events that pose a threat to cultural heritage. Due to the catastrophic nature of fire events and the heightened number of incidences within historic buildings (Landis 2017), fire often features within many risk and vulnerability assessments for cultural heritage (Ashley 2013, Salazar et al. 2021, Uluç Keçik 2022). The availability of statistics regarding the frequency of fires is relatively good in some places around the world, with potential incidence rates for different levels of prevention, mitigation, and control measures also beginning to be defined (Tétreault 2008). However, there are currently no publicly available fire risk statistics that are finely tuned to museums in the UK.

**Table 1:** Example of adverse events, how they are characterised in the model, and the logic associated with them.

Process Description	Fraction Affected (Range)	Condition Loss (Range)	Mean Time (Range)	Logic
Serious fire where fire service is called in several rooms	0.06–0.2	0.6–1	200–600 years	The fraction affected value reflects that these objects are within several rooms, rather than the entire collection. The condition loss value will be high in the case of a fire. The mean time value is for a museum with medium fire prevention, mitigation, and control measures, estimated by statistics within Kidd (2002) Kincaid (2022), Tétreault (2008), and Landis (2017).
Serious incident of heritage crime where high value item is stolen	0.006–0.02	0.6–1	20–60 years	The fraction affected value reflects that a single high value item is likely to only contribute a small proportion to the overall value of a collection. The condition loss value will be high in the case of stolen objects (assuming that they are not recovered). The mean time value is for a museum with a medium crime prevention, mitigation, and control measures, estimated by statistics within Coupe & Kaur (2005), Welsh & Farrington (2009), Bradley et al. (2012), and Tseloni et al. (2018).
Flood from overflowing drains affecting several ground rooms	0.06–0.2	0.06–0.2	60–200 years	The fraction affected value reflects that these objects are within a several rooms, rather than the entire collection. The condition loss value is reflective of the fact that potentially only a part of the value of the object may be lost under these circumstances. The mean time value is suggestive of a museum that has a medium surface water flood risk according to GOV.UK (2025).
Roof leak in heavy rain causing damage to collections	0.006–0.02	0.006–0.02	Once per year	The fraction affected value reflects that these events likely happen in very confined spaces. The condition loss value reflects that only a small part of the value of each object would likely be lost under these circumstances. The mean time value is suggestive of a museum that has few water ingress events each year.

Like fire, the theft of objects can also constitute another form of total value loss to an artifact since, in the words of the National Trust Manual of Housekeeping, “a stolen object is, to all intents and purposes, as lost as one that is destroyed in a fire" (National Trust 2011). The high incidence of heritage crime (Bradley et al. 2012) means that security threats are another focus of risk assessments for cultural heritage (Brokerhof et al. 2017). However, more sector-specific statistics need to be developed to understand the risk levels for museums with different combinations of crime prevention measures.

Flooding is a common concern for heritage managers due to the physical damage and staining caused by exposure to water, as well as the mould infestations that often emerge after an event, and has been included within, or made the focus of, many risk and vulnerability assessments (D’Ayala et al. 2020, Gandini et al. 2020, Miranda and Ferreira 2019, Ogden 2012). The flood risk of a heritage asset is closely aligned to the proximity of a site to bodies of water, as well as the drainage capacity of local land and wastewater pipes.

Water ingress from a leaking roof (or rainwater goods), generating staining and humidity problems, is also a common occurrence, though exactly how often these events happen on average and how much damage is sustained per incident has not been investigated. The substantial number of projects within the MEND and PBIF funding programmes focused upon the replacement of faulty roofs suggests that the deterioration of these elements is a frequent issue experienced within historic buildings, many of which have highly complex roof arrangements comprised of a range of historic materials, making them vulnerable to damage (Cassar and Pender 2003). The incidences of flooding and water ingress are likely to increase in many places around the world as climate change exacerbates the number of intense rainfall events (Martel et al. 2021, Orr et al. 2018) and storm surges (Bevacqua et al. 2020).

The short list of specific risk scenarios presented in Table 1 are only a very limited selection of all the different types of hazards that a museum, gallery, archive or library has to contend with. To build a fully comprehensive model, a much more expansive collection of hazards (and associated metrics), e.g., earthquake, pests, terrorism, or breakages, associated with specific risk scenarios would have to be included.

### Modelling time to failure with the Weibull distribution

At every time step of the simulation, an adverse event can occur or not. A way to model this is to sample a time before an adverse event from a distribution centred around the mean time (based on the A score of the ABC risk framework) and sample the time to the next event from a probability distribution. To that end, we adopt the Weibull distribution. This probability distribution is commonly used in reliability engineering and survival analysis (Elmahdy 2015, O’Connor 2011) to model the failure rates of mechanical and electronic systems, among other applications. While the random mean time introduces variability, the Weibull distribution adds a crucial layer of probabilistic modelling. This combination ensures that the simulation is both flexible and realistic, accurately reflecting the complexity of real-world event timing.

An event-driven simulation could also be a suitable method, in particular for cases where degradation is dominated by prominent and infrequent adverse events rather than continuous processes. However, there are several benefits of the chosen approach. Continuous degradation is a key component of the model, described via a simple differential equation that is updated at every time step. While this could be incorporated into an event-driven framework, the aim is to build a platform that can accommodate more complex continuous dynamics in the future, including interactions between concurrent degradation processes, for which a time-stepped approach is more natural and scalable. It is also important to prepare the model for the eventual inclusion of time-dependent policies, for example, a conservation protocol that only occurs between two dates, or even a gradual change of temperature caused by climate change. These processes are more intuitively and transparently applied to a time-stepped framework. Finally, managing a long event queue in an event-driven simulation with extensive lists of adverse events may be computationally intensive. Some events may be relatively high frequency (hundreds of times a year) while some are very low (once in a few hundred years). As a result, the simulation would still effectively process most time-steps.

The Weibull distribution is characterised by two parameters: *λ*, the scale parameter, and *k*, the shape parameter. The probability density function (PDF) of the Weibull distribution is given by:

$$f(x;\lambda ,k)=\left\{\begin{array}{ll}\frac{k}{\lambda }{\left(\frac{x}{\lambda }\right)}^{k-1}{e}^{-{(x/\lambda )}^{k}}\quad &x\ge 0\\ 0\quad &x < 0\end{array}\right.$$

In this equation, *x* represents the random variable (e.g., the time to an adverse event), *λ* represents the scale parameter, which determines the characteristic mean time before an adverse event, and *k* represents the shape parameter, which determines the shape of the distribution.

- For *k* < 1, the probability of an event decreases as time progresses.
- For *k* = 1, the probability of an event remains constant over time.
- For *k* > 1, the probability of an event initially increases with time and then eventually decreases. This characteristic is known as the “bathtub curve" and is commonly observed in reliability engineering.

**Fig. 3:**
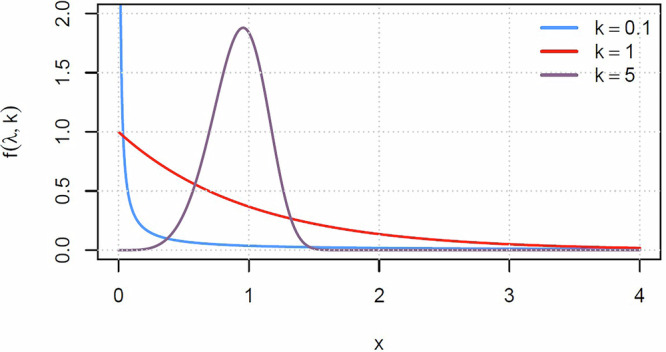
Graphical representation of the probability density function of the Weibull distribution for different values of k and *λ* = 1.

The effect of k can be seen in Fig. 3. The scale parameter of the plotted Weibull distributions is 1, but this parameter takes different values in the simulation. The implementation of the model demonstrated here uses *k* = 1 (H5). A Weibull distribution with a scale parameter of 200 years implies that an event, for example, a fire, happens with a mean time between events of 200 years, and a median time between events of 138.6 years. This means that it is actually more likely that an event will happen before 200 years than after, specifically there is a 63.2% chance that the event will happen before 200 years, and 36.8% afterwards.

It is conceivable that in some well known hazards in heritage *k* > 1. For example, the older a book is, the more likely a piece will break during handling. However, this and similar scenarios where this might hold have never been measured.

The bathtub curve produced when *k* > 1 makes sense in an engineering context, but it remains to be seen if the same logic applies in heritage systems. The idea is that, initially, the probability of an event increases with time because the failure rate accelerates due to wear-out mechanisms. This is often seen in engineering applications in the early life of a product when there is a higher likelihood of defect due to manufacturing imperfections or stress on components. As time progresses, the probability of an event peaks and then declines. This decline occurs because as weaker components fail early in the life of the system, the surviving components tend to be more robust, leading to a decrease in the failure rate. This phase is often referred to as the “random failures" period, where failures occur due to random events rather than wear-out mechanisms.

### Monte Carlo sampling to determine the impact of adverse events

Within the model, the B and C risk parameters of the ABC framework are used to simulate the impact of the adverse events and update the condition of affected agents:

- When an adverse event occurs, a function is called to select a random subset of agents based on the fraction of the collection affected parameter. This function determines the number of agents affected by the event.
- For each affected agent, their condition is updated by subtracting a random value within the specified range of the extent of impact on the condition of objects parameter. This simulates the degradation or damage caused by the event to the affected agents.

This way of proceeding is usually referred to as Monte Carlo sampling. The properties are assumed to be normally distributed and that the ranges listed in the ABC model correspond to 95% confidence intervals (H6).

### Description of the model steps

All the features listed below are combined together in an agent-based model that simulates the behavior of a population of agents over time. The main steps of the model are as follows:

1. **Initialisation:** The model initialises the population of agents with their initial conditions. Each agent has a condition *C* that follows a normal distribution with a mean and standard deviation given by the user, capped between 0 and 100. Information on the current condition of the collection can be introduced in this step.
2. **Simulation Loop:** The model iterates over multiple years, simulating the behavior of the population for each year.
  21. **Continuous Degradation:** For each year, the condition of each agent is decreased by a degradation rate, calculated from Temperature (T) and Relative Humidity (RH) conditions.
  22. **Adverse Events:** The model predicts the occurrence of adverse events using a Weibull distribution. For each year, a random mean time before an adverse event is generated, and a time until event occurrence is sampled from the Weibull distribution. If the sampled time is less than or equal to 1 year, an adverse event is simulated for a fraction of the population, reducing their condition accordingly.
  23. **Percentage of Objects in Good Condition:** After each year, the model calculates the percentage of objects in good condition (condition *C* > 0) and records it.
3. **Repetition:** The simulation is repeated multiple times to capture the variability of the system. Only 10 runs are usually enough to reveal a characteristic pattern of decay for the collection.
4. **Analysis:** After all simulations are completed, the model analyses the results, including histograms of initial and final conditions, time series of the percentage of objects in good condition, and presenting an overall collection lifetime calculated as the average time to reach 1% of the agents in good condition.

### Implementation in R

Table 2 describes the main inputs and outputs of the first implementation of the model. The code is mostly reproducible following the information given in the previous sections, except for some non-trivial decisions. The truncnorm package generates random numbers from a truncated normal distribution. It is used to initialise the conditions of the agents, ensuring that the starting values are within a realistic and predefined range (i.e., that no agent has a condition above 1 or below 0).

**Table 2:** User Inputs and Main Outputs.

Category	Parameter	Description
**Inputs**	num_agents	Number of agents (population size)
	num_years	Number of years to simulate
	num_simulations	Number of simulation runs
	lower_bound	Lower bound of the initial condition distribution
	upper_bound	Upper bound of the initial condition distribution
	mean	Mean of the initial condition distribution
	sd	Standard deviation of the initial condition distribution
	deg_processes	List of degradation processes with parameters
	T	Temperature for degradation rate calculation
	RH	Relative humidity for degradation rate calculation
	pH	pH level for degradation rate calculation
	DP0	Initial degradation potential for degradation rate calculation
	DP1	Estimated end of life for degradation rate calculation
**Outputs**	all_conditions	Final conditions of agents for each simulation
	all_percentage_good	Percentage of agents in good condition over time
	time_to_1_percent	Time taken for the percentage of objects in good condition to drop to 1%
	average_time	Average time to reach 1% good condition across all simulations
	sd_time	Standard deviation of the time to reach 1% good condition
**Plots**	hist	Histogram of initial and final agent conditions
	time_series_plot	Time series plot of percentage of objects in good condition

For each year in the simulation, a time until the next event occurrence is sampled from a Weibull distribution with shape parameter 1 (indicating an exponential distribution) and scale parameter equal to the mean time between adverse events specified by the users.

The following R code snippet illustrates the use of the ‘rweibull’ function to sample the time until the next event occurrence:

mean_time <- runif(1, process$mean_time[1], process$mean_time[2])

time_until_event <- rweibull(1, shape = 1, scale = mean_time)

In this snippet, mean_time is a random mean time before an adverse event sampled from the specified range. This accounts for the uncertainty in the time to failure. The rweibull function then generates a random deviate from a Weibull distribution with shape parameter 1 and scale parameter equal to the sampled mean time. This sampled time until event occurrence determines whether an adverse event will happen in the current year.

## Example results

To generate some example results, the model was run using the input parameters described in Tables 1 and 3. Since there is little available data within the field for initialisation, calibration, or validation of the model, i.e., empirical data from long-term controlled experiments and historical degradation reports (producing a time series of condition), this exercise can be considered as a toy application to illustrate the outputs that the model produces and how they can be visualised. The value of longitudinal experiments and the generation of ‘epidemiological’ datasets, i.e., quantitative evidence of patterns of decay in collections, is something that the field is reckoning with, but it will be some time before the results are readily available for comparison. Hence, it is not possible to know whether the model results reproduce domain knowledge because there is little codified knowledge on the subject. In this sense, the publication of this model is timely and can encourage the initiation of possible validation datasets. While the toy application demonstrated here is for paper, which has the most advanced damage function, other mathematical models of material change are close to being ready for use in this way.

**Table 3:** The parameters used for the generation of example model results.

Input	Parameter value	Logic
num_agents	1000	Sensitivity analysis suggests that 1000 agents is the minimum number to produce reliable model results.
num_years	1500	This is a suitable time frame for the model results.
num_simulations	10	Sensitivity analysis suggests that 10 simulation runs are satisfactory when testing the model with only four adverse events.
lower_bound	0	The lower bound of the initial conditions should reflect that some objects have already reached end of life.
upper_bound	100	The higher bound of the initial conditions should reflect that some objects are in optimum condition.
mean	70	Reflects that the majority of the collection is in a reasonable condition.
sd	20	Reflects the considerable variation in condition within a collection.
deg_processes	Paper	We employ the most advanced damage function to test the model here, but it could be expanded to include damage functions for other materials in future.
T	20 °C	A commonly used reference condition for the control of temperature within collections.
RH	50%	A commonly used reference condition for the control of relative humidity within collections.
pH	7	A neutral pH that minimises acid hydrolysis is assumed for this demonstration.
DP0	3000	A DP of 3000 is often considered the maximum value for paper.
DP1	300	For paper, a DP value of around 300 is often considered to mark the end of its useful life.

Note that the parameters relating to the adverse events can be found in Table 1.

**Fig. 4:**
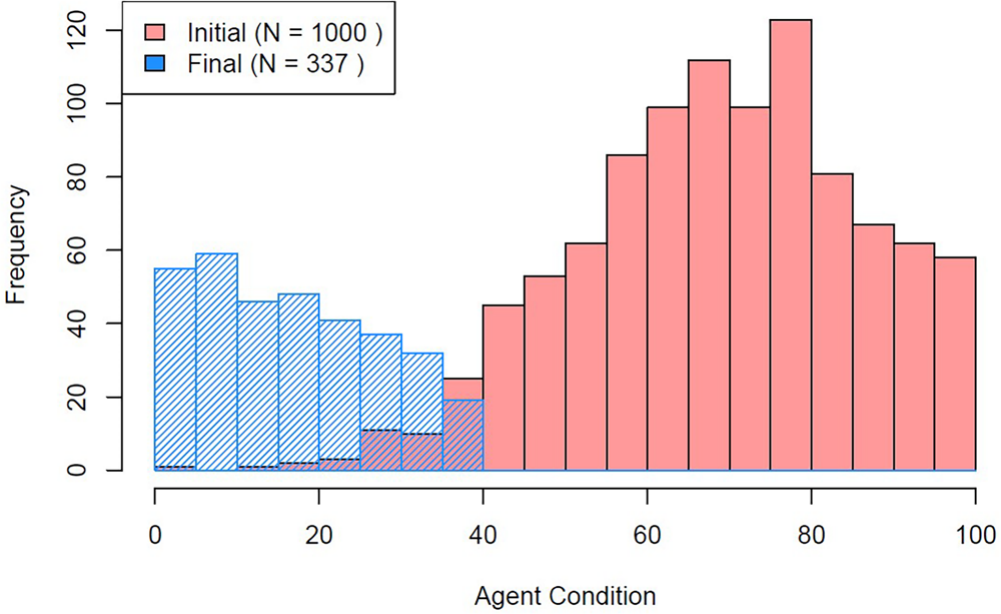
Histogram of collection condition before and during a simulation.

The main outcome of the model is the decay in condition of the collection as a consequence of all the combined degradation processes. Figure 4 shows two histograms of condition, at the start of the process and after 500 years. The mean condition of the collection gradually displaces to the left. When an object reaches a contingent condition of 0, it “falls off" from the simulation (it is deaccessioned, conserved, or otherwise receives some action or investment).

**Fig. 5:**
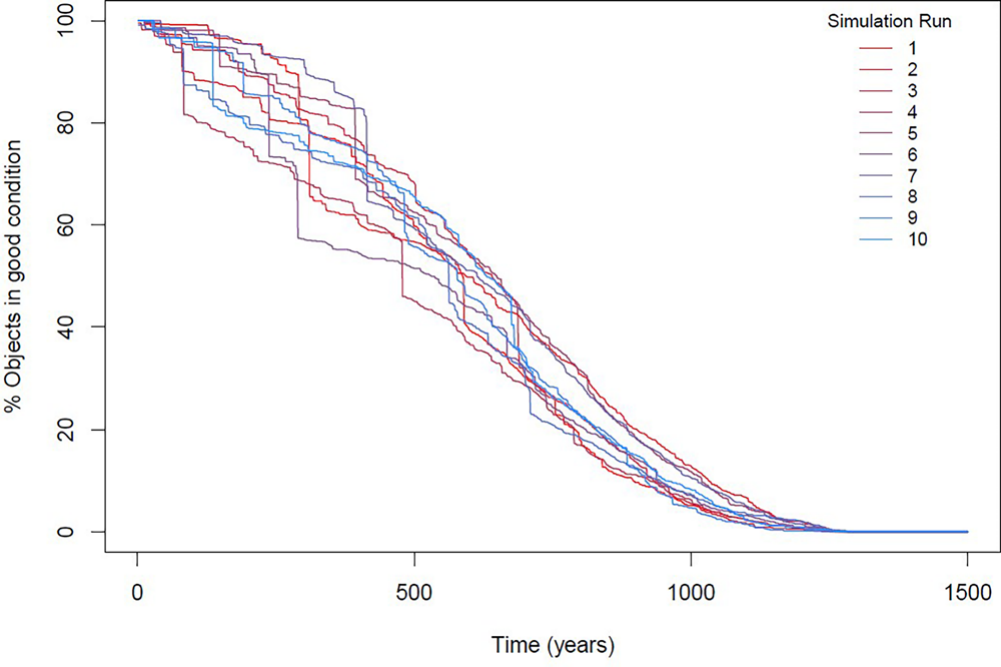
Graph demonstrating simulation example of 10 model runs. Some runs exhibit large impact risks while others show many low impact risks, yet overall behaviors are very similar.

At every time step, the model counts how many objects remain in good condition. This allows visualisations like Fig. 5, which shows the evolution of the percentage of objects in good condition over time for 10 example simulation runs. A simulation run represents one possible future lifetime, marked by a series of random accidents. The model should run as many times as necessary to express all the alternative futures for a collection. While nothing prevents us from studying hundreds or thousands of futures, it is interesting to note that in practice, 10 runs are already enough to cover most of the variability in outcomes. Models with more degradation processes may require more runs.

The decay dynamics shown in Fig. 5 have some interesting features. The most important observation is that the collection degradation pathways tend to display similar shapes and timelines. This convergence happens even if some futures are more unlucky than others. For example, simulation run 6 experiences several fires, while simulation run 1 experiences only minor accidents. And yet, the final outcome, and the overall lifetime, are not as different as could be expected. This occurs because an accumulation of small accidents can be as destructive as a few large accidents. Note that this emergent behaviour is caused only by a list of 4 adverse events (Table 1).

The non-linearity of the decay patterns is due mostly to the initial distribution of conditions. Some non-linearity is also caused by the underlying damage function that continuously erodes the condition. In other implementations of the model, further non-linearity could be introduced by adding self-reinforcing degradation processes (for example, when something is a bit broken it breaks more easily, or when acid degradation starts, it accelerates). Such effects would have an effect on the shape of the distribution of conditions, which would change during the simulation.

One strength of this model is the ability to compare the consequences of degradation processes, regardless of whether they are probabilistic or continuous. One way to do this is what modellers call an “ablation study". In other words, removing one factor at a time in order to observe the effect on the overall outcome. Table 4 compares different scenarios where degradation processes have been removed. In this scenario, removing chemical degradation has the biggest effect, more than tripling the lifetime. On the other hand, preventing fires only makes a small difference to the lifetime. Of course, these results are only as good as the input data. We should not conclude from this that fire is the least destructive process to collections. Rather, we should use this evidence to critically evaluate our estimate of fire risk. Have we underestimated its frequency, impact or capacity to reduce value? This is how this method may be helpful to fine-tune the the outcomes of an ABC risk assessment: by allowing us to visualise the consequences of our estimates.

**Table 4:** Collection Lifetime, Standard Deviation of Lifetime, and Max Lifetime under different conditions.

Condition	Collection Lifetime (time to reach 1%)	Standard Deviation of Lifetime	Max Lifetime
All degradation processes	1025.35	27.56	1062
Without chemical degradation*	3715.1	418.04	4803
Without fire	1051.1	28.33	1080
Without theft*	1052.1	16.58	1073
Without flooding*	1077.9	17.06	1102
Without leaking*	1182.5	42.79	1237

Rows marked with an asterisk (*) show a statistically significant difference compared to “All degradation processes".

The model also doubles as an uncertainty propagation analysis. Because risks are defined with ranges, rather than a single value, the model outputs a spectrum of possible outcomes. The standard deviations included in Table 4 are produced by averaging the results of all the simulation runs. This helps identify in which cases the effect of a risk is not statistically significant. In this example, removing fire does not result into a statistically significant improvement on the lifetime.

## Extensions to the model

### Incorporate diverse degradation functions

A number of damage functions are developed enough to be added to the model. However, its transferability is still limited for many diverse collections due to the lack of advanced damage functions for different material types. In the future, it can be updated to include new research and thus it has the ability to become a living database of all known damage functions, making it an increasingly realistic in its simulation of heritage lifetimes. The interaction between different degradation mechanisms can be modelled to account for synergistic effects. For example, chemical degradation might make materials more susceptible to physical wear, or high humidity could exacerbate both biological and chemical degradation processes.

There are also likely interactions between objects within a collection that are related to the distribution of different materials and their specific characteristics. For instance, a collection with a lot of plastic will have high levels of off-gassing, which, if not adequately managed could generate high degradation rates of other materials. The same could be said for some probabilistic risks, for example, a store of cellulose nitrate provides a significant fuel source that, if ignited, could lead to more extensive fire damage within a museum. It is feasible that these types of interactions might also be included when the model is in a very advanced state of development.

### Dynamic environmental conditions

Rather than relying on static values for parameters like temperature, relative humidity, the model can incorporate variable environmental data. Seasonal variations can be introduced to simulate the cyclical nature of environmental conditions, which is especially interesting if mechanical damage is added.

### Agent-based model enhancements

**Fig. 6:**
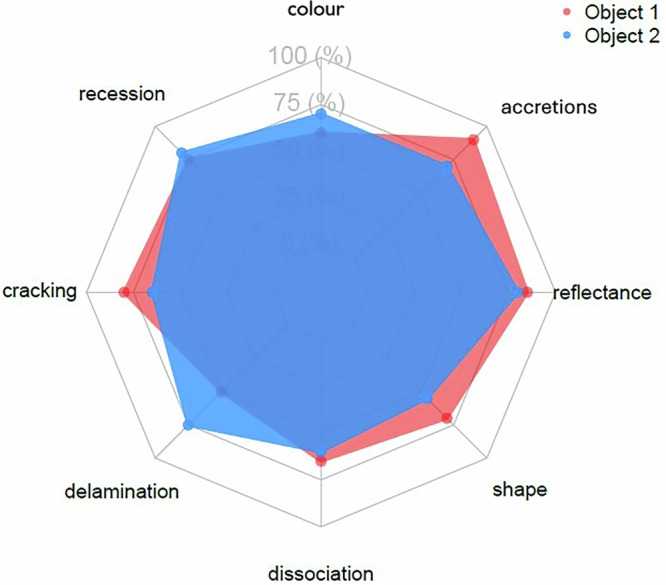
Spider diagram illustrating an example of how the model can include different pathways towards condition reduction.

The first step to enhance the agent-based model involves increasing the heterogeneity among agents. In particular, each agent can degrade in a different way. Furthermore, it is possible to define several properties for each agent. Figure 6 shows an invented example, inspired by the spider plots used for sensory profiles, i.e., in wine tasting. In this case, each separate agent has 8 dimensions of condition. All of them can be lost, and the agent would become “unacceptably degraded" when one of them reaches the threshold. The second enhancement to the model would be to add interactions between agents. Early experimentation with this idea (Duran Casablancas et al. 2024) has involved the interaction between book-agents and visitor-agents, which causes accelerated degradation due to handling. A third enhancement would be to consider sub-spaces within collections, such as different rooms or storage areas.

### Scenario planning

Scenario planning would extend the model’s applicability by exploring potential future scenarios and their impacts on the collection. This involves running simulations under different assumptions, such as varying climate conditions, funding levels for conservation, or changes in storage environments. Initial research has involved evaluating the cost of delaying the decision to deacidify a collection (Duran-Casablancas et al. 2021).

## Implications for future research

Can the principles of Collections Demography be extended to cover any heritage typology? The answer depends on the testing of six hypotheses, which sustain the model presented in this paper:

- H1 Threshold Existence: There exist identifiable thresholds for unacceptable change in a wide diversity of heritage typologies. This could be difficult to define for heritage typologies that have complex or diverse social uses.
- H2 Sharp Response: The perception and response to condition change in heritage objects increase sharply beyond a certain threshold. If this hypothesis does not hold, the definition of lifetime will be more arbitrary.
- H3 Proportional Degradation: An absolute condition can be defined in a way that is directly proportional to one or more key measurable properties of heritage objects (e.g., color, strength). This could be complicated in degradation processes which lead to multi-dimensional phenomena that is not characterised with a single metric, such as crack networks.
- H4 Risk Forecasting: The ABC risk assessment model or similar frameworks can evolve into a quantitative tool for forecasting risks associated with heritage objects. This is achievable with a combination of data and expertise.
- H5 Time Distribution: A Weibull distribution effectively models the probability of adverse events or failure rates of heritage objects. Exploring this requires data.
- H6 Modelling Uncertainty: The uncertainty in the severity of adverse events is measurable and can be modelled, for example with a normal distribution.

The model presented can be useful even before these six hypothesis are thoroughly investigated (or indeed even if one or two are disproved). The uses of the model are:

- To compare the consequences of probabilistic and continuous degradation.
- To assess critically the quality of risk assessment estimations, by checking if the long-term impact of estimated risks is realistic in comparison with other processes.
- To study the propagation of different types of uncertainty to the final lifetime estimation. For example, comparing the uncertainty caused by measurement errors (e.g. ± 3% RH) with the uncertainty caused by expert estimations of unknown parameters.

While the six hypotheses are necessary for a *universal* model, there is a high potential to use this approach in specific contexts. We know this works for paper collections. What other collection types could benefit from this type of analysis with our current level of knowledge?

## Data Availability

We do not analyse or generate any datasets, because our work proceeds within a theoretical and mathematical approach. The R implementation of the model described in this document is available on GitHub: https://github.com/jgraubove/generaldegradation.
